# Urinary metabolic signatures reflect cardiovascular risk in the young, middle-aged, and elderly populations

**DOI:** 10.1007/s00109-020-01976-x

**Published:** 2020-09-11

**Authors:** Paula J. Martinez, Marta Agudiez, Dolores Molero, Marta Martin-Lorenzo, Montserrat Baldan-Martin, Aranzazu Santiago-Hernandez, Juan Manuel García-Segura, Felipe Madruga, Martha Cabrera, Eva Calvo, Gema Ruiz-Hurtado, Maria G Barderas, Fernando Vivanco, Luis M Ruilope, Gloria Alvarez-Llamas

**Affiliations:** 1 Department of Immunology, Immunoallergy and Proteomics Laboratory, IIS-Fundación Jiménez Díaz, UAM, Avenida Reyes Católicos 2, 28040 Madrid, Spain; 2 CAI-RMN, Universidad Complutense de Madrid, Madrid, Spain; 3 Department of Vascular Physiopathology, Hospital Nacional de Parapléjicos SESCAM, Toledo, Spain; 4 Department of Biochemistry and Molecular Biology I, Universidad Complutense, Madrid, Spain; 5 Departament of Geriatrics, Hospital Virgen del Valle, SESCAM, Toledo, Spain; 6 Ibermutuamur, Madrid, Spain; 7 Cardiorenal Translational Laboratory, Instituto de Investigación I+12, Hospital Universitario 12 de Octubre, Madrid, Spain; 8 CIBER-CV, Hospital Universitario 12 de Octubre, Madrid, Spain; 9 School of Doctoral Studies and Research, Universidad Europea de Madrid, Madrid, Spain; 10 REDINREN, Madrid, Spain

**Keywords:** Biomarkers, Cardiovascular risk, Elderly, Early prevention, Lifetime risk, Metabolomics

## Abstract

**Abstract:**

The predictive value of traditional cardiovascular risk estimators is limited, and young and elderly populations are particularly underrepresented. We aimed to investigate the urine metabolome and its association with cardiovascular risk to identify novel markers that might complement current estimators based on age. Urine samples were collected from 234 subjects categorized into three age-grouped cohorts: 30–50 years (cohort I, young), 50–70 years (cohort II, middle-aged), and > 70 years (cohort III, elderly). Each cohort was further classified into three groups: (a) control, (b) individuals with cardiovascular risk factors, and (c) those who had a previous cardiovascular event. Novel urinary metabolites linked to cardiovascular risk were identified by nuclear magnetic resonance in cohort I and then evaluated by target mass spectrometry quantification in all cohorts. A previously identified metabolic fingerprint associated with atherosclerosis was also analyzed and its potential risk estimation investigated in the three aged cohorts. Three different metabolic signatures were identified according to age: 2-hydroxybutyrate, gamma-aminobutyric acid, hypoxanthine, guanidoacetate, oxaloacetate, and serine in young adults; citrate, cyclohexanol, glutamine, lysine, pantothenate, pipecolate, threonine, and tyramine shared by middle-aged and elderly adults; and trimethylamine N-oxide and glucuronate associated with cardiovascular risk in all three cohorts. The urinary metabolome contains a metabolic signature of cardiovascular risk that differs across age groups. These signatures might serve to complement existing algorithms and improve the accuracy of cardiovascular risk prediction for personalized prevention.

**Key messages:**

• Cardiovascular risk in the young and elderly is underestimated.

• The urinary metabolome reflects cardiovascular risk across all age groups.

• Six metabolites constitute a metabolic signature of cardiovascular risk in young adults.

• Middle-aged and elderly adults share a cardiovascular risk metabolic signature.

• TMAO and glucuronate levels reflect cardiovascular risk across all age groups.

**Electronic supplementary material:**

The online version of this article (10.1007/s00109-020-01976-x) contains supplementary material, which is available to authorized users.

## Introduction

Cardiovascular disease (CVD) is the leading cause of premature death worldwide despite continual improvements in primary and secondary prevention. The predominant underlying pathology of CVD is atherosclerosis, a chronic and systemic immunoinflammatory disease of medium- and large-sized arteries. Formation of an atheroma plaque and progressive arterial obstruction takes place silently and asymptomatically, which in many cases results in a sudden event with potentially fatal consequences. Because cardiovascular risk is multifactorial and includes genetic and environmental factors, different algorithms have been developed to estimate cardiovascular risk in apparently healthy persons in the short-medium term (5–10 years), and these are based mainly on age, sex/gender, race, cholesterol levels, blood pressure, smoking habits, and the presence of diabetes. The majority of individuals with low cardiovascular risk over the next 10 years, however, show high risk in the long term, which can be calculated over their likely remaining lifetime (lifetime risk calculation or LTR QRISK) [1]. The impact of traditional risk factors for cardiovascular events changes with age [2, 3], and thus currently available estimators in all age groups may be inappropriate [4, 5], particularly, when considering that they are typically developed in middle-aged subjects.

Cardiovascular risk is especially underestimated in young adults. Consequently, few of them reach treatment thresholds for intervention, and prevention strategies are delayed. There is evidence for subclinical coronary atherosclerosis in this population [6], and although the presence of atheroma plaque *per se* might not serve to estimate cardiovascular risk, the extent of coronary artery disease rather than individual plaque lesions and their vulnerability to rupture can be considered evidence of subclinical risk [7, 8]. Healthy lifestyle changes made early in adulthood are known to decrease the risk of cardiovascular events later in life [9], clearly supporting the view that prevention strategies should start early. Thus, novel tools to improve cardiovascular risk assessment are needed to better stratify young individuals and more precisely define whom to target for personalized intervention. At the other extreme, old age is a major risk factor for CVD; however, individuals of the same chronological age may differ considerably with respect to their overall health status, thus limiting the predictive capacity of chronological age alone in determining overall disease risk [10]. Indeed, conventional cardiovascular risk estimators underestimate survival in the elderly [11], and this can result in overmedication [12].

Omics technologies are powerful tools in biomarker discovery and validation, *via* the identification of significant variations in the abundance of proteins or metabolites without the preselection of molecular targets. The metabolome reflects the ultimate response of an organism to a (physio)pathological condition and provides an integrated profile, or signature, of biological status and metabolic health. Accordingly, defining the chemical phenotypes of health or disease using metabolomics is gaining attraction in cardiovascular risk stratification [13–15]. In a longitudinal study of elderly (mean age at baseline, 71 years) with or without CVD, the simultaneous addition of biomarkers of cardiovascular and renal abnormalities was found to substantially improve the risk stratification for death from cardiovascular causes beyond that of a model based only on established risk factors [16]. Similarly, it was recently shown that a metabolomic signature characterized largely by intermediates of fatty acid oxidation improved the prediction of cardiovascular events in the elderly [17]. Using metabolomics approaches, we previously identified specific metabolic fingerprints in urine reflecting the development of atherosclerosis and acute coronary syndrome/patient recovery [18] and the cardiovascular risk of subjects undergoing coronary artery bypass surgery [19]. By proteomics, we recently identified a urinary cardiovascular risk signature in young adults based on six proteins [20].

In the present study, we sought to investigate the metabolic alterations associated with cardiovascular risk in three independent cohorts stratified by age. Specifically, we aimed to identify metabolic cardiovascular risk markers in young adults (30–50 years old) and then to evaluate the identified metabolic patterns of risk in two older populations—middle-aged (50–70 years) and elderly (> 70 years) cohorts.

## Methods

### Patient selection and urine collection

This is a cross-sectional study of 234 subjects clinically categorized into the following three cohorts stratified by age: cohort I, 30–50 years; cohort II, 50–70 years; and cohort III, > 70 years (Table 1). Each cohort was subclassified into three groups reflecting the presence of cardiovascular risk and considering age-dependent population characteristics: “control” (C) group, “risk factor” (RF) group, and “cardiovascular event” (CVE) group. As the prevalence of traditional cardiovascular risks factors is low in cohort I, the C group included apparently healthy subjects without medication, whereas the RF group included individuals with estimated glomerular filtration rate (eGFR) < 100 mL/min/1.73 m^2^ or albuminuria and at least one of the following conditions: arterial hypertension (or on antihypertensive medication), hyperglycemia (blood glucose > 110 mg/dL), and/or metabolic syndrome. In cohort II, the C group included individuals without traditional cardiovascular risk factors and with blood pressure in the high-normal range according to the 2013 ESH/ESC European guidelines (≥130/85 mm Hg), whereas the RF group included individuals with blood pressure in the high-normal range and eGFR of 20–60 mL/min/1.73 m^2^ or albuminuria. In cohort III, the C group included subjects with eGFR > 60 mL/min/1.73 m^2^, and the RF group included individuals with eGFR of 20–60 mL/min/1.73 m^2^. In all three cohorts, the CVE group included individuals who had had a stroke or acute myocardial infarction in the previous 3 years (Table 1). All subjects included in the study underwent a detailed medical history interview, physical examination, and biochemical profile. Lifetime risk or LTR QRISK (thereafter referred to as LTR in the manuscript) was estimated using the lifetime cardiovascular risk calculator (https://qrisk.org/lifetime/).

**Table 1:** Baseline clinical data of different aged-based cohorts expressed as mean ± SD or percentages. Young adults (30–50 years), middle-aged (50–70 years), and elderly (> 70 years)

	Young adults (discovery)			Young adults (confirmation)			Middle-aged			Elderly		
	C	RF	CVE	C	RF	CVE	C	RF	CVE	C	RF	CVE
*n*	12	10	12	33	25	25	28	23	25	25	25	25
eGFR (mL/min/1.73 m^2^)	92 ± 9	95 ± 19	98 ± 27	93 ± 10	89 ± 15	98 ± 21	81 ± 10	75 ± 20	77 ± 18	75 ± 13	40 ± 9	66 ± 19
DM %	0	10	0	0	12	8	4	44	28	40	32	40
Age (years)	42 ± 6	45 ± 6	45 ± 6	43 ± 5	44±5	45 ± 4	60 ± 5	62 ± 5	61 ± 5	83 ± 5	86 ± 5	83 ± 6
Sex (male), %	50	40	67	49	72	80	75	91	68	52	48	48
Total cholesterol (mg/dL)	193 ± 29	211 ± 38	158 ± 36	193 ± 37	210 ± 37	147 ± 40	180 ± 21	171 ± 24	157 ± 37	144 ± 29	145 ± 35	145 ± 26
Triglycerides (mg/dL)	86 ± 46	140 ± 110	121 ± 72	82 ± 38	190 ± 99	143 ± 158	109 ± 39	131 ± 65	126 ± 56	110	88 ± 37	103 ± 43
HDL (mg/dL)	69 ± 18	54 ± 15	43 ± 10	71 ± 17	45 ± 14	41 ± 10	54 ± 14	51 ± 16	52 ± 18	44 ± 15	45 ± 14	41 ± 11
LDL (mg/dL)	108 ± 31	132 ± 32	90 ± 33	105 ± 32	135 ± 33	81 ± 38	105 ± 22	95 ± 21	80 ± 35	79 ± 23	70 ± 20	78 ± 22
Glycemia (mg/dL)	80 ± 7	100 ± 41	100 ± 24	80 ± 8	97 ± 31	107 ± 43	102 ± 13	123 ± 24	118 ± 40	104 ± 25	110 ± 44	113 ± 44
Creatinine (mg/dL)	0.9 ± 0.1	0.81 ± 0.13	0.9 ± 0.2	0.81 ± 0.12	0.90 ± 0.12	0.90 ± 0.18	0.9 ± 0.2	1.1 ± 0.3	1.0 ± 0.3	0.8 ± 0.2	1.4 ± 0.3	1.0 ± 0.4
Uric acid (mg/dL)	4.9 ± 1.4	5.1 ± 1.0	5.7 ± 1.4	4.6 ± 1.2	6.3 ± 1.7	5.8 ± 1.4	6.2 ± 1.4	6.4 ± 1.7	6.1 ± 1.4	4.9 ± 1.2	8 ± 2	5.7 ± 1.9
SBP	113 ± 9	130 ± 8	122 ± 16	113 ± 9	135 ± 12	122 ± 19	136 ± 11	141 ± 14	143 ± 16	140 ± 23	131 ± 21	150 ± 27
DBP	73 ± 8	84 ± 9	75 ± 10	71 ± 8	88 ± 9	76 ± 12	80 ± 8	81 ± 9	84 ± 11	76 ± 15	71 ± 15	77 ± 14
LTR QRISK	24 ± 7	34 ± 8	-	23 ± 6	42 ± 10	-						

*C* control group, *CVE* cardiovascular event group, *DBP* diastolic blood pressure, *DM* mellitus diabetes, *eGFR* estimated glomerular filtration rate, *HDL* high-density lipoprotein, *LDL* low-density lipoprotein, *LTR QRISK* lifetime risk, *SBP* systolic blood pressure, *RF* cardiovascular risk factor group

A spot urine sample was collected from each participant in a sterile container. Samples were centrifuged at 16,200×*g* for 10 min, and supernatants were collected and stored at – 80 °C until analysis. The study was approved by the Ethics Committee of Ibermutuamur, Hospital 12 de Octubre, and Hospital del Valle, as appropriate, and was conducted according to the principles of the Declaration of Helsinki. All patients signed written informed consent before inclusion.

### Metabolite analysis by NMR

In a first discovery phase, we aimed to identify novel metabolites linked to cardiovascular risk in urine samples from cohort I (*n* = 34), using ^1^H nuclear magnetic resonance (NMR) as described [18, 21] (Table 1). Briefly, an aliquot of 300 μL of urine samples was diluted (1:1) in 200 mmol/L sodium phosphate buffer in D_2_O containing 0.01 mmol/L sodium trimethylsilyl propionate for chemical shift referencing. NMR analysis was performed at 278 K on a Bruker 700 MHz AVANCE III instrument equipped with a 5-mm triple resonance, z axis gradient cryoprobe. Spectra were processed using TOPSPIN (v3.2, Bruker BioSpin) and analyzed with AMIX software (v3.6.8, Bruker BioSpin). Each spectrum was partitioned into small spectral regions of 0.04 ppm (buckets). Normalization was performed based on the total intensity of the spectrum. The distribution of every bucket over the ensemble of spectra was evaluated by AMIX software for the significance analysis of variables (buckets) using a confidence level of 90%. Those discriminating buckets (*p* value < 0.05) resulting from the statistical comparison of the CVD groups were further considered for identification. The human metabolome database (HMDB version 4.0) and Chenomx NMR Suite 8.30 profiler (Chenomx) were used for theoretical identification [22]. Two-dimensional NMR analysis, including homonuclear correlation spectroscopy ^1^H–^1^H (COSY), total correlated spectroscopy (TOCSY), and heteronuclear single-quantum correlation spectroscopy (^1^H–^13^C HSQC), was used for unequivocal identification in our particular samples.

### Mass spectrometry target analysis

Confirmation of the variations in metabolites according to cardiovascular risk was accomplished by mass spectrometry (MS) target analysis in selected reaction monitoring (SRM) mode using all urine samples of cohort I (*n* = 83) (Table 1). Those metabolites showing abundance changes linked to cardiovascular risk were also analyzed in cohorts II and III. A 6460 Triple Quadrupole LC-MS/MS (1200 Series, Agilent Technologies) controlled by Mass Hunter Software (v4.0 Agilent Technologies) was used as described [18, 19, 21]. Briefly, a 100 μL sample of urine was used for analysis, and proteins were removed by organic precipitation. Metabolite separation took place at 0.4 mL/min in an acetonitrile gradient for 5 min in positive or negative mode. Optimal conditions of analysis were established by analyzing commercial metabolite standards (Online Resource 1). Peak areas were used for intergroup comparison.

### Testing a CVD metabolic fingerprint for cardiovascular risk estimation

The following metabolites, previously identified by our group associated with atherosclerosis development and cardiovascular risk, were also analyzed in the three cohorts (*n* = 234): citrate, cyclohexanol, glucuronate, glutamine, guanidoacetate, lysine, oxaloacetate, malate, pantothenate, pipecolate, serine, threonine, trimethylamine N-oxide (TMAO), 1-methylhydantoin, and tyramine [18, 19, 21]. Analysis was carried out by SRM as described above.

### Statistical analysis

For the NMR analysis, the discriminating buckets were obtained by AMIX software using significance analysis of variables (90% confidence level) and Welch’s ANOVA. Statistical analysis of SRM data was performed using the Mann-Whitney nonparametric test, applying ROUT method to detect outliers (setting Q to 5%), by GraphPad Prism (v6.01, GraphPad Prism Software). Univariate and multivariate receiver operating characteristic (ROC) curves were generated by Monte Carlo cross-validation (MCCV) using balanced subsampling on the Metaboanalyst web server. In each MCCV, two-thirds (2/3) of the samples are used to evaluate the feature importance, and the top important features are then used to build classification models that are then validated on the remaining 1/3 of the samples. This procedure was repeated multiple times to calculate the performance and confidence interval of each model. Random forests was selected as the feature ranking built-in method for features selection. Though the area under the curve values (AUC) may be overestimated as they are not derived from independent sample, we calculated them for additional comparison between the proposed models. Correlation between metabolites abundance and estimated LTR values was investigated using Spearman’s test.

## Results

We analyzed the metabolic profile of urine samples with the aim of identifying novel indicators of cardiovascular risk. A second aim was to determine whether the alterations found in young adults are also evident in the middle-aged and elderly. Baseline characteristics of the different study population cohorts are shown in Table 1.

### Metabolic alterations linked to cardiovascular risk in young, middle-aged, and elderly populations

**Fig. 1:**
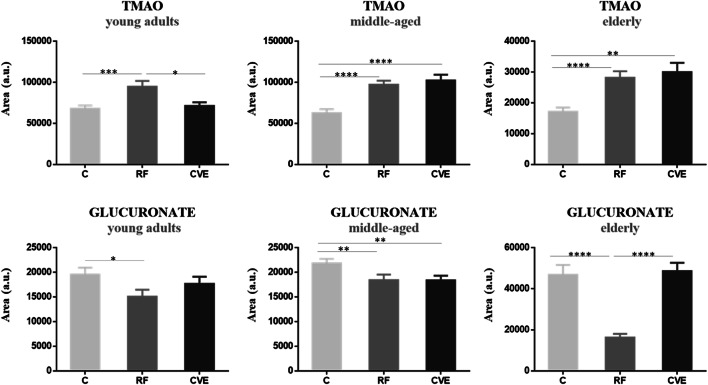
Urinary TMAO and glucuronate reflect cardiovascular risk in young adults, middle-aged, and elderly adults. Variation in metabolite abundance variation in the three age-based cohorts investigated (30–50 years, 50–70 years, and > 70 years) is shown. Differences in abundance between control (C), cardiovascular risk factor (RF), and cardiovascular event (CVE) groups are represented as mean ± SEM. *p value < 0.05; **p value < 0.01; ***p value < 0.001; ****p value < 0.0001 (Online Resource 2)

Of all the metabolites identified by NMR analysis, only TMAO and glucuronate showed significant changes in abundance according to cardiovascular risk in all three aged-based cohorts. The same trend of alteration was observed between the RF group and the C group for both metabolites, with TMAO significantly higher and glucoronate significantly lower in the RF group. By contrast, the trend for TMAO and glucuronate in the CVE group was more heterogeneous across the cohorts (Fig. 1).

### A urinary metabolic pattern is associated with cardiovascular risk specifically in young adults

**Fig. 2:**
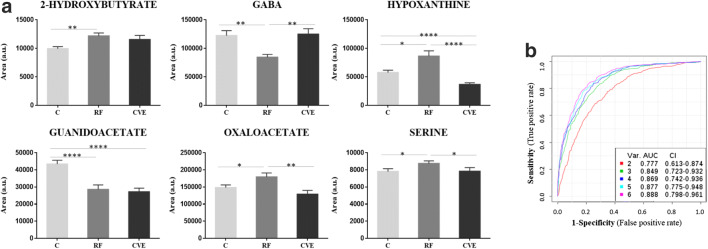
Cardiovascular risk metabolic signature in young adults. Panel **a** shows the variation in metabolite abundance between control (C), cardiovascular risk factor (RF), or cardiovascular event (CVE) groups in young adults (30–50 years) represented as mean ± SEM. *p value < 0.05; **p value < 0.01; ****p value < 0.0001 (Online Resource 3). Panel **b** shows receiver operating characteristic curves including area under the curve (AUC) values

In the first discovery phase by NMR in the young population, we identified several novel metabolites potentially associated with cardiovascular risk (discovery cohort, Table 1). Three metabolites were further confirmed by SRM, showing significant changes in levels between the RF and C groups in a confirmation cohort of young adults (Table 1): 2-hydroxybutyrate and hypoxanthine (higher) and gamma-aminobutyric acid (GABA) (lower) (Fig. 2a). We also analyzed urine metabolites that we previously identified in CVD [18, 19] using SRM in the confirmation cohort, finding that guanidoacetate abundance was significantly lower in the RF group than in the C group, whereas the opposite was seen for oxaloacetate and serine (Fig. 2a). We calculated ROC curves for the six individual metabolites, finding that the combination of all six yielded the best performance with an AUC value of 0.888 (Fig. 2b). We then assessed whether these metabolic alterations were also evident in middle-aged and elderly subjects. None of the six metabolites showed the same trend of variation in the other two populations investigated (cohorts II and III).

**Fig. 3:**
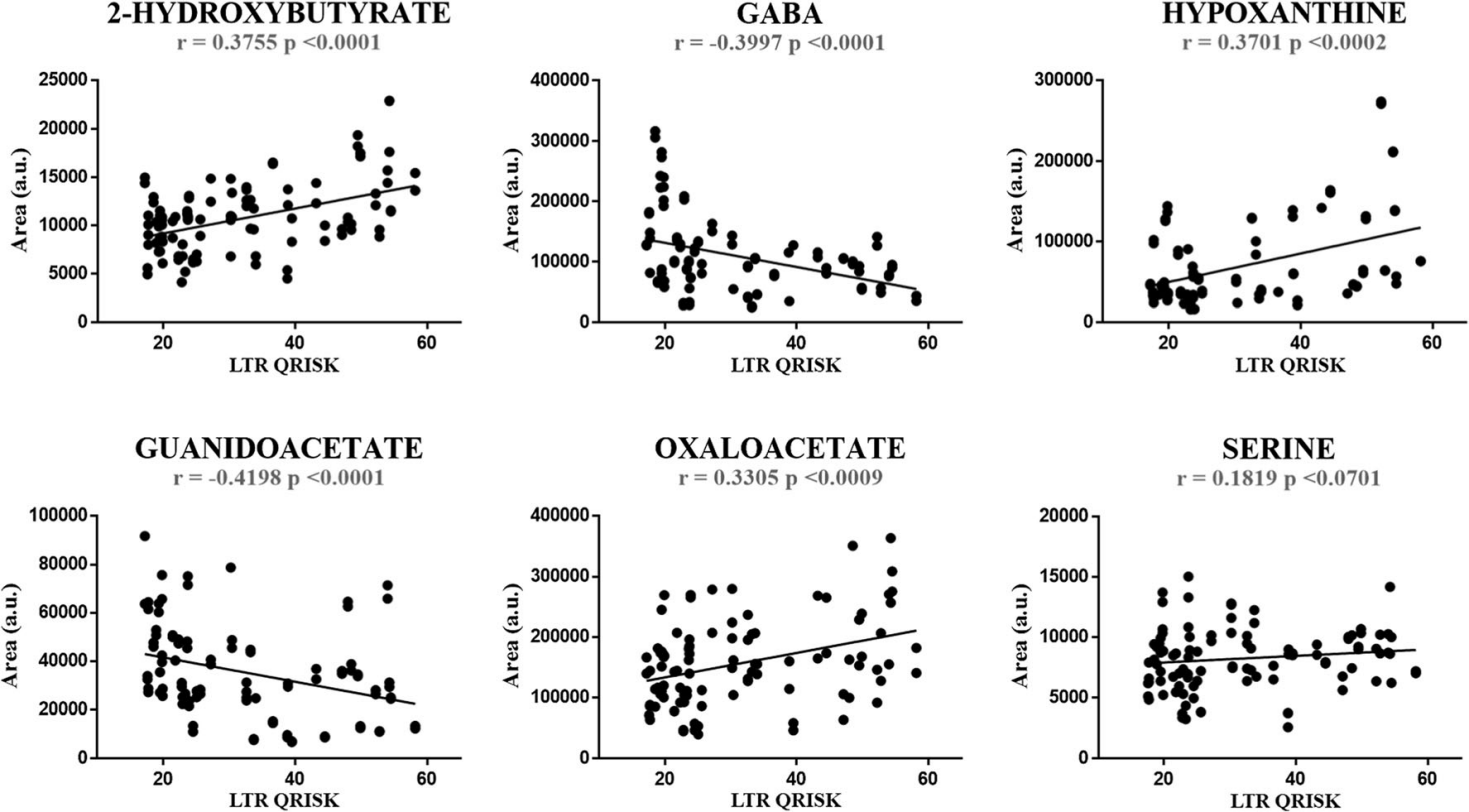
Correlation between metabolite abundance and lifetime risk. Lifetime risk was estimated using LTR QRISK®, and Spearman correlation was performed. *p value < 0.05; **p value < 0.01; ****p value < 0.0001

We next examined for potential correlations between the levels of the identified metabolites in urine and the LTR score evaluated in young adults, finding significant correlations for all with the exception of serine (Spearman correlation values: 2-hydroxybutyrate, *r* = 0.3755 *p* < 0.0001; hypoxanthine, *r* = 0.3701 *p* < 0.0002; oxaloacetate, *r* = 0.3305 *p* < 0.0009; guanidoacetate, *r* = − 0.4198 *p* < 0.0001; GABA, *r* = − 0.3997 *p* < 0.0001) (Fig. 3).

### Middle-aged and elderly subjects share a common metabolic feature linked to cardiovascular risk

**Fig. 4:**
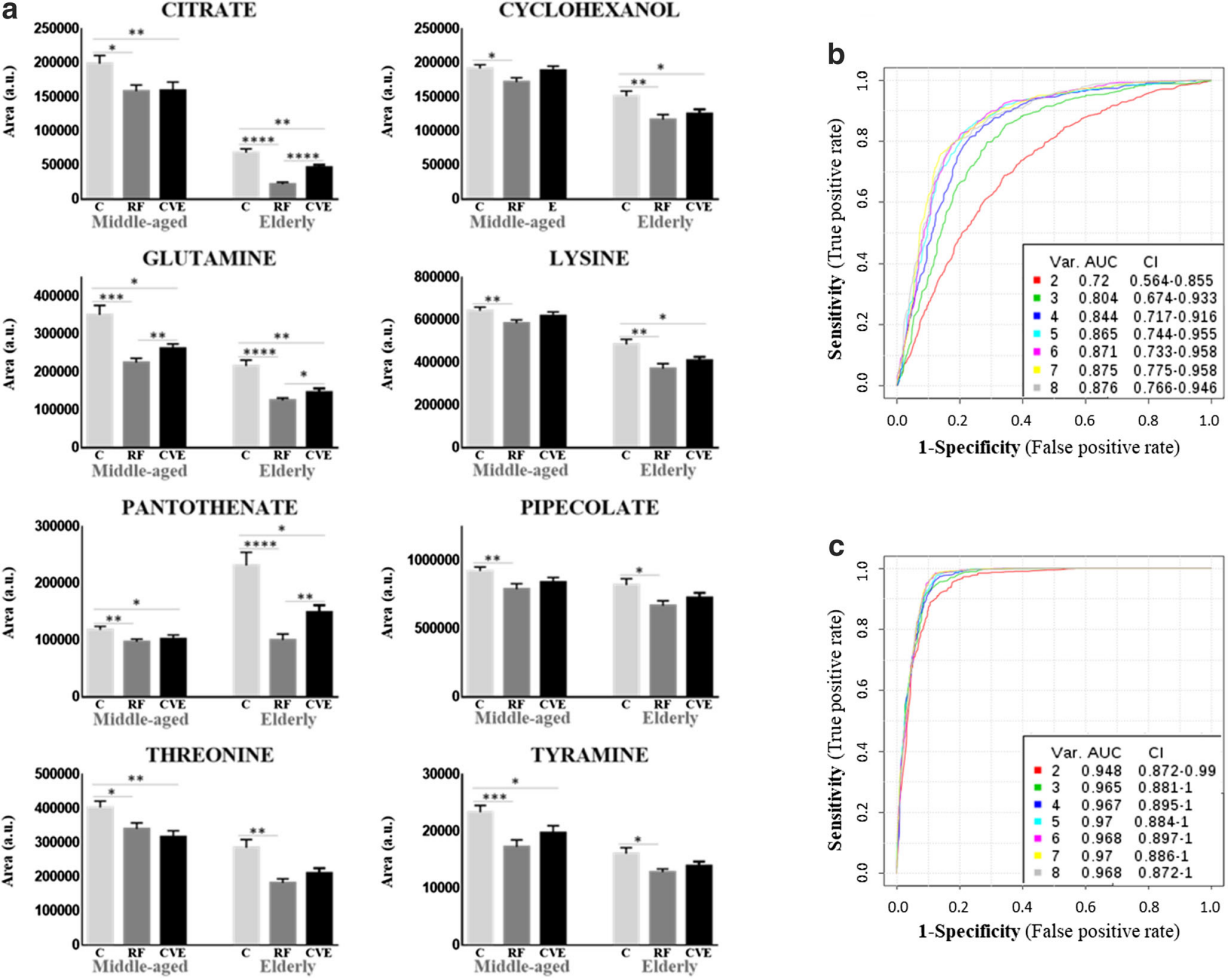
Cardiovascular risk metabolic signature shared by middle-aged and elderly populations. Panel **a** shows variation in metabolite abundance between control (C), cardiovascular risk factor (RF), or cardiovascular event (CVE) groups in middle-aged (50–70 years) and elderly (> 70 years) cohorts, represented as mean ± SEM (**a**). *p value < 0.05; **p value < 0.01; ***p value < 0.001; ****p value < 0.0001 (Online Resource 4). Panels **b** and **c** show receiver operating characteristic (ROC) curves including area under the curve (AUC) values for cohorts II and III, respectively

We identified a specific metabolic signature in cohorts II and III without evident variation in young adults. Levels of citrate, cyclohexanol, glutamine, lysine, pantothenate, pipecolate, threonine, and tyramine were lower in the RF group than in the C group in both cohorts (Fig. 4a). The sensitivity and specificity of metabolites were evaluated in cohorts II and III separately (Fig. 4b, c). ROC curve analysis showed that in cohort II, the best performance was obtained when the eight metabolites were combined, with AUC values from 0.72 to 0.876 (Fig. 4b), and the added value of sequentially including metabolites in the model was clear. In cohort III, the performance of the model did not change as dramatically, varying from 0.948 to 0.968 when 2 or 8 metabolites were combined, respectively (Fig. 4c), and with and individual AUC value for citrate of 0.942. Additionally, two metabolites showed an altered profile in the cardiovascular risk factor group uniquely in the elderly: malate (lower) and 1-methylhydantoin (higher).

## Discussion

In the present study, we report differences in the urinary metabolome associated with cardiovascular risk. We additionally identified specific metabolic signatures in age-based cohorts.

### Cardiovascular risk in the young population: oxidative stress underlies main alterations in urinary metabolites

**Fig. 5:**
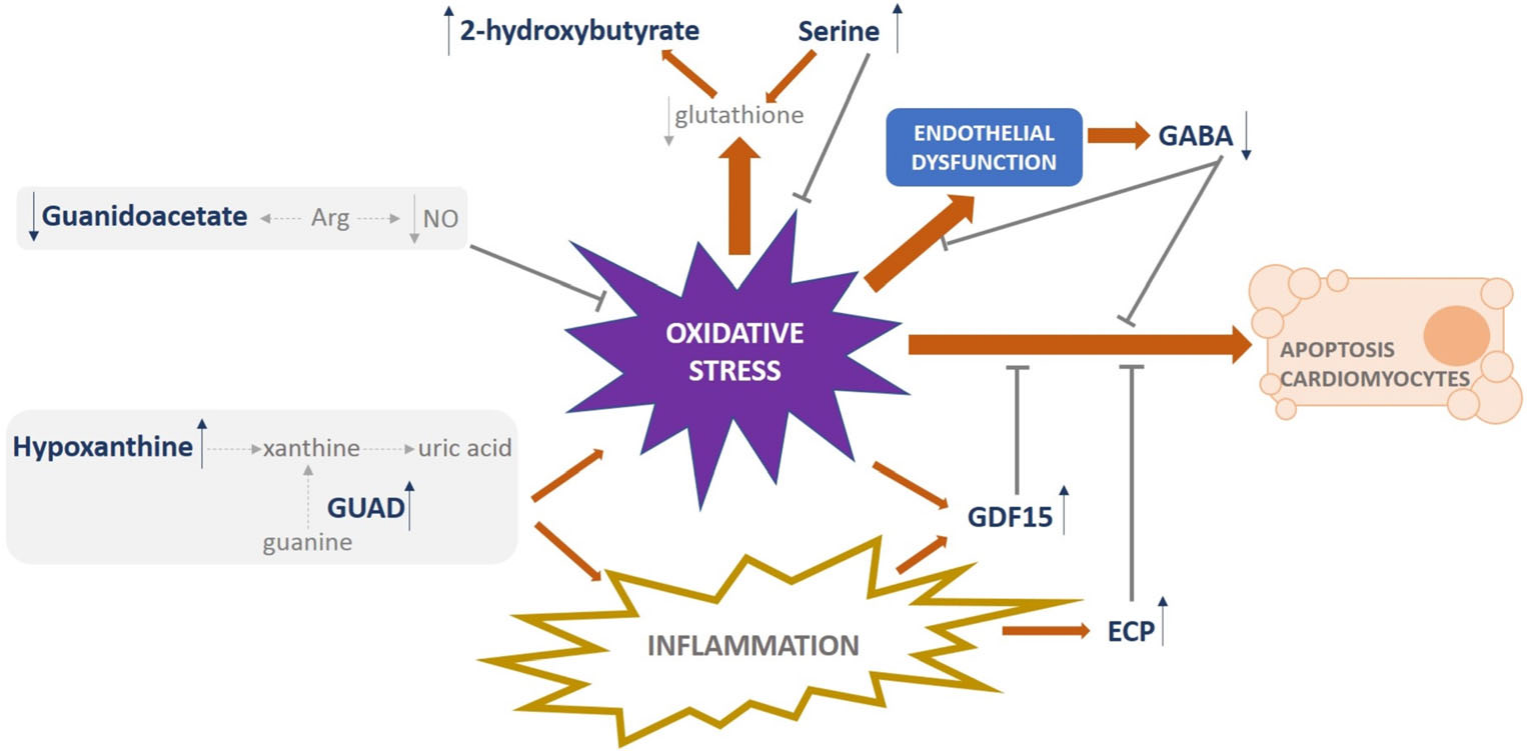
Cardiovascular risk is reflected in urine by metabolic regulation under oxidative stress conditions in young adults. Purine metabolites precursors of uric acid (hypoxanthine and GUAD), intermediates in glutathione synthesis (2-hydroxybutyrate and serine), and molecules involved in counteracting oxidative stress, endothelial dysfunction, or cardiomyocytes apoptosis (guanidoacetate, GABA, GDF15, and ECP) are shown as the main molecular players reflecting cardiovascular risk in urine. Bold letter represents identified urinary metabolites and proteins showing altered levels associated with cardiovascular risk. Arrows represent higher (↑) or lower (↓) variation. *ECP* eosinophil cationic protein, *GABA* gamma-aminobutyric acid, *GDF15* growth differentiation factor 15, *GUAD* guanine deaminase, *NO* nitric oxide

Oxidative stress is known to be involved in the pathogenesis of CVD [23–25], and prior metabolomics studies in human urine point to an important contribution for oxidative stress in acute coronary syndrome [26] and hypertension in persons < 50 years [27]. In the same line, our previous proteomic study highlighted oxidative stress as a major functional category that is altered by cardiovascular risk factors already present in young adults [20]. Our present metabolomic study, performed in the same cohort, also provides clear evidence for the existence of metabolic deregulation under conditions of oxidative stress and points to specific targets that can be easily monitored (Fig. 5). Hypoxanthine induces endothelial dysfunction through ROS production and oxidative stress-induced apoptosis [28]. We found higher urinary levels of hypoxanthine associated with cardiovascular risk factors. Hypoxanthine is converted to xanthine, which in turn is converted to uric acid, and an increase in these three purine metabolites has been reported in response to cardiac ischemia [29]. The conversion of guanine to xanthine in the uric acid cycle is performed by the aminohydrolase guanine deaminase (GUAD) [30]. Interestingly, GUAD was one of the main urinary proteins found altered (higher) in the presence of cardiovascular risk factors in our previous proteomics study [20], altogether supporting a potential role for hypoxanthine and GUAD as biomarkers of CVD early in life and also the contribution of uric acid—the final produce of purine metabolism—as a key determinant in cardiovascular risk, previously described [31]. We also found higher levels of 2-hydroxybutyrate with increased cardiovascular risk, in agreement with the reported elevated levels in patients with microvascular ischemic heart disease [32]. 2-Hydroxybutyrate is an early biomarker of insulin resistance and impaired glucose regulation, and higher levels might be related to increased lipid oxidation and oxidative stress [33]. We previously found higher serum levels of 2-hydroxybutyrate in patients with an acute coronary syndrome [34]. Additionally, urinary secretion of 2-hydroxybutyrate reflects shifts in the rate of glutathione synthesis. In this context, we previously found diminished levels of glutathione in the atherosclerotic aorta [19], and here, we associate higher levels of this metabolite with an increase in the demand for glutathione in oxidative stress conditions. Of note, serine has previously been shown to decrease oxidative stress while supporting glutathione synthesis [35, 36], in line with our observations here (higher in the RF group) and supporting a compensatory mechanism in conditions of reduced glutathione levels. By contrast, subjects with cardiovascular risk showed lower levels of guanidoacetate, a trend previously observed in patients with hypertension and further aggravated by albuminuria or diabetes [21, 37]. Arginine is a main precursor of nitric oxide (NO) and guanidoacetate and has antioxidant properties [38]. Levels of NO are reduced in an oxidative environment, pointing also to reduced levels of arginine, which would be in accord with the observed lower levels of guanidoacetate. We also found that GABA, a metabolite synthesized and released by endothelial cells, was lower in subjects with cardiovascular risk, which might be explained in terms of its protective function by inhibiting ROS generation and monocyte adhesion [39]. A hypothesis has been established on an inhibitory role for GABA in atherosclerosis [40], acting as a potential urinary marker of cardiovascular risk when it is diminished, which is in line with our previous observations in GDF15 (growth differentiation factor 15) and ECP (eosinophil cationic protein) [20].

All of the altered metabolites with the exception of serine significantly correlated with LTR: 2-hydroxybutyrate, hypoxanthine, and oxaloacetate positively and guanidoacetate and GABA negatively, supporting their potential added value in assessing cardiovascular risk when estimated along the lifetime.

### The cardiovascular risk metabolic signature differs with age

Changes in the dynamics of biological processes during aging and their influence over cardiovascular risk are to be expected, and traditional cardiovascular risk factors tend to have less impact in older individuals or even show the opposite behavior, such as the inverse relationship between body mass index and coronary artery calcification [41]. Indeed, the pathophysiology of CVD in the elderly may be different from that of younger people [42]. Along this line, there is evidence supporting the central role of mitochondrial oxidative stress, mitochondrial damage, and biogenesis, in addition to crosstalk between mitochondria and cellular signaling in cardiac and vascular aging [43].

The metabolites found altered in cohorts II and III represent a cardiovascular risk urinary signature that appears later in life. Interestingly, the trend in the RF group was more pronounced in people > 70 years than in those aged 50–70 years for most metabolites, pointing to an aggravated response with age. This is further supported by the lack of variation in people aged 30–50 years.

All of the metabolites examined here showing alteration in their levels, with the exception of threonine, also displayed altered levels in our previous studies in CVD with the same trend as reported in the present study. For instance, the levels of cyclohexanol, glutamine, lysine, pipecolate, and tyramine were lower in the urine and aortic tissue of rabbits fed a cholesterol-rich diet to induce atherosclerosis [18]; citrate was lower in the urine from patients with chronic kidney disease (CKD) with associated CVD [44]; and pantothenate showed the same trend in hypertensives *versus* non-hypertensive subjects, further aggravated by albuminuria [21]. Supporting our findings, levels of urinary lysine and tyramine levels show lower levels in patients with the metabolic syndrome patients (aged between 24–72 years) and correlate negatively with cardiometabolic features and inflammatory biomarkers [45, 46]. Also, decreased levels of glutamine in urine have been identified as a hyperlipidemia biomarker (aged 25–65 years) [47].

### TMAO, a robust urinary biomarker of cardiovascular risk at any age

TMAO and glucuronate levels were significantly altered in those individuals with cardiovascular risk independently of the age range investigated. These observations further underpin the utility of TMAO as a potential biomarker of CVD, supporting previous evidence of its association with a higher incidence of mortality in patients with CKD, peripheral arterial disease, diabetes mellitus, or heart failure [48, 49]. It has also been reported that higher circulating levels of TMAO may independently predict the risk of subsequent cardiovascular events and mortality [48], and consequently, TMAO is a good candidate for incorporation into existing risk stratification tools [49]. Indeed, analysis of urine TMAO levels in > 1000 patients with myocardial infarction revealed a 2.2-fold increase compared with control subjects [50]. Our data show that higher levels of urinary TMAO are associated with cardiovascular risk before a cardiovascular event occurs. In young adults, TMAO levels in the CVE group were not significantly different to those in the C group, and this was also the case for GABA, hypoxanthine, oxaloacetate, and serine, thus uniquely reflecting cardiovascular risk itself. However, middle-aged and elderly individuals who experienced a cardiovascular event had higher levels of TMAO than the respective C groups. This observation might indicate that elderly individuals have a limited capacity to recover from organ damage after an acute cardiovascular event [51]. Guanidoacetate or 2-hydroxybutyrate might also reflect established organ damage in young adults, as altered levels were also observed in the CVE group.

## Limitations

The present study fulfilled the requirements of an omics study in terms of group size and technical workflow [52]; however, one limitation was the relatively low number of patients from a clinical perspective, and further studies are warranted in larger cohorts before considering the use of these new urinary biomarkers in clinical practice. Differences in medication resulting from lifestyle and CVD prevention strategies adjusted individually might have influenced the observed data. Even so, the identified metabolic signatures may still have value in addition to current estimators once the individual is being controlled by available therapy. That being said, the strength of the correlations found in this study and the fact that the metabolic variations reported here confirm previous observations from our group and others linked to cardiovascular risk raise the possibility that the required larger cohorts would simply confirm our data. Future prospective trials with clinical cardiovascular endpoints would be needed to address whether the metabolites shown here can complement traditional risk factors.

## Conclusions

We identified three different metabolic signatures in urine associated with cardiovascular risk according to age: specific to young adults (30–50 years), shared by middle-aged (50–70 years), and elderly (> 70 years) adults and common to all three age-based cohorts. These data confirm previous findings on specific biomarkers and provide novel molecular indicators to be evaluated for lifetime risk of cardiovascular disease. The metabolic signatures identified differ between those individuals with cardiovascular risk and control subjects. A trend towards control values after overcoming a cardiovascular event could be observed mainly in young adults, indicating potentially better cardiovascular recover with pharmacological treatment in this population.

## Electronic supplementary material

ESM 1 (DOCX 23 kb)
