# Supplementary material for: Urinary metabolic signatures reflect cardiovascular risk in the young, middle-aged, and elderly populations
Source: J Mol Med (Berl). 2020 Sep 11;98(11):1603–13. doi: 10.1007/s00109-020-01976-x (PMC7591416; doi:10.1007/s00109-020-01976-x)
Supplement: Supplementary file 1 — (DOCX 23 kb) [file 109_2020_1976_MOESM1_ESM.docx]

SUPPLEMENTARY MATERIAL

Online Resource 1. SRM analysis conditions for metabolites of interest.

| **Metabolite** | **m/z value (precursor→fragmentor)** | **Fragmentor Voltage (v)** | **CE (v)** | **Ionization Mode** |
| --- | --- | --- | --- | --- |
| 1-methyldantoin | 113.1→69.1 | 90 | 10 | (+) |
| 2-hydroxybutyrate | 103.1→57.1 | 120 | 10 | (-) |
| Citrate | 191.1→86.9 | 120 | 14 | (-) |
| Cyclohexanol | 101.2→84 | 130 | 10 | (+) |
| Glucuronate | 193.1→112.9 | 120 | 10 | (-) |
| GABA | 104.1→45.2 | 60 | 22 | (+) |
| Guanidoacetate | 118.1→76.1 | 120 | 10 | (+) |
| Hypoxanthine | 137.1→55.2 | 100 | 34 | (+) |
| Lysine | 147.1→130 | 130 | 10 | (+) |
| Serine | 105.9→60.1 | 175 | 20 | (+) |
| Glutamine | 147.15→84.1 | 84 | 18 | (+) |
| Threonine | 120.1→102.9 | 130 | 18 | (+) |
| Oxalacetate | 133.08→61.1 | 156 | 30 | (+) |
| Pantothenate | 220.4→90.1 | 80 | 14 | (+) |
| Pipecolate | 130.2→84.1 | 72 | 14 | (+) |
| TMAO | 76.1→59 | 175 | 22 | (+) |
| Tyramine | 138.2→121.1 | 60 | 10 | (+) |

CE: Collision energy, GABA: Gamma-aminobutyric acid, TMAO: Trimethylamine N-oxide.

**Online Resource 2. p-values of each group-comparison Figure 1:** control (C), cardiovascular risk factor (RF) or cardiovascular event (CVE) groups.

|  | **C vs RF** | **CVE vs RF** | **CVE vs C** |
| --- | --- | --- | --- |
| **GLUCURONATE** |  |  |  |
| **young adult** | 0.0384 | 0.0784 | 0.5914 |
| **middle-aged** | 0.0089 | 0.8827 | 0.0065 |
| **elderly** | 4.441882e-08 | 3.639627e-11 | 0.4624 |
| **TMAO** |  |  |  |
| **young adult** | 0.0005 | 0.012 | 0.4314 |
| **middle-aged** | 4.029501e-07 | 0.9496 | 0.000032 |
| **elderly** | 0.000038 | 0.8217 | 0.0012 |

TMAO: Trimethylamine N-oxide.

**Online Resource 3. p-values of each group-comparison Figure 2:** control (C), cardiovascular risk factor (RF) or cardiovascular event (CVE) groups.

|  | **C vs RF** | **CVE vs RF** | **CVE vs C** |
| --- | --- | --- | --- |
| **2-HYDROXYBUTYRATE** | 0.0015 | 0.1608 | 0.3426 |
| **GABA** | 0.0052 | 0.0024 | 0.7198 |
| **HYPOXANTHINE** | 0.0255 | 0.000001 | 0.000075 |
| **GUANIDOACETATE** | 0.000013 | 0.9165 | 0.000002 |
| **OXALOACETATE** | 0.0441 | 0.0017 | 0.0501 |
| **SERINE** | 0.0205 | 0.0372 | 0.8514 |

GABA: Gamma-aminobutyric acid

**Online Resource 4. p-values of each group-comparison Figure 4:** control (C), cardiovascular risk factor (RF) or cardiovascular event (CVE) groups.

|  | **C vs RF** | **CVE vs RF** | **CVE vs C** |
| --- | --- | --- | --- |
| **CITRATE** |  |  |  |
| middle-aged | 0.0118 | 0.585 | 0.00093 |
| elderly | 1.019283e-11 | 6.988844e-07 | 0.002 |
| **CYCLOHEXANOL** |  |  |  |
| middle-aged | 0.0322 | 0.1294 | 0.448 |
| elderly | 0.0041 | 0.4931 | 0.0391 |
| **GLUTAMINE** |  |  |  |
| middle-aged | 0.0002 | 0.0053 | 0.0345 |
| elderly | 0.000007 | 0.0421 | 0.0028 |
| **LYSINE** |  |  |  |
| middle-aged | 0.0056 | 0.2641 | 0.1264 |
| elderly | 0.0021 | 0.2905 | 0.0478 |
| **PANTOTHENATE** |  |  |  |
| middle-aged | 0.0091 | 0.9446 | 0.0388 |
| elderly | 0.000005 | 0.0055 | 0.161 |
| **PIPECOLATE** |  |  |  |
| middle-aged | 0.0018 | 0.2309 | 0.0528 |
| elderly | 0.0249 | 0.226 | 0.2138 |
| **THREONINE** |  |  |  |
| middle-aged | 0.0369 | 0.6587 | 0.0076 |
| elderly | 0.0011 | 0.1811 | 0.0575 |
| **TYRAMINE** |  |  |  |
| middle-aged | 0.0004 | 0.1389 | 0.0225 |
| elderly | 0.0457 | 0.2514 | 0.2355 |
